# The D Domain of LRRC4 anchors ERK1/2 in the cytoplasm and competitively inhibits MEK/ERK activation in glioma cells

**DOI:** 10.1186/s13045-016-0355-1

**Published:** 2016-11-25

**Authors:** Zeyou Wang, Qin Guo, Rong Wang, Gang Xu, Peiyao Li, Yingnan Sun, Xiaoling She, Qiang Liu, Qiong Chen, Zhibin Yu, Changhong Liu, Jing Xiong, Guiyuan Li, Minghua Wu

**Affiliations:** 1 The Key Laboratory of Carcinogenesis of the Chinese Ministry of Health, Xiangya Hospital, Central South University, Changsha, Hunan 410008 China; 2 Cancer Research Institute, The Key Laboratory of Carcinogenesis and Cancer Invasion of the Chinese Ministry of Education, Central South University, Changsha, Hunan 410008 China; 3 Hunan Key Laboratory of Nonresolving Inflammation and Cancer, Disease Genome Research Center, The Third Xiangya Hospital, Central South University, Changsha, Hunan 410013 China; 4 Department of Laboratory Medicine, The Second Xiangya Hospital, Central South University, Changsha, Hunan 410011 China; 5 Medical College, University of South China, Hengyang, Hunan 421001 China; 6 Hunan Provincial Tumor Hospital and the Affiliated Tumor Hospital of Xiangya Medical School, Central South University, Changsha, 410013 Hunan China; 7 Department of Pathology, The Second Xiangya Hospital, Central South University, Changsha, Hunan 410011 China; 8 Department of Ophthalmology, Xiangya Hospital, Central South University, Changsha, Hunan 410008 China

**Keywords:** Leucine-rich repeat, D domain, CD domain, ERK1/2, MAPK

## Abstract

**Background:**

As a well-characterized key player in various signal transduction networks, extracellular-signal-regulated kinase (ERK1/2) has been widely implicated in the development of many malignancies. We previously found that Leucine-rich repeat containing 4 (LRRC4) was a tumor suppressor and a negative regulator of the ERK/MAPK pathway in glioma tumorigenesis. However, the precise molecular role of LRRC4 in ERK signal transmission is unclear.

**Methods:**

The interaction between LRRC4 and ERK1/2 was assessed by co-immunoprecipitation and GST pull-down assays in vivo and in vitro. We also investigated the interaction of LRRC4 and ERK1/2 and the role of the D domain in ERK activation in glioma cells.

**Results:**

Here, we showed that LRRC4 and ERK1/2 interact via the D domain and CD domain, respectively. Following EGF stimuli, the D domain of LRRC4 anchors ERK1/2 in the cytoplasm and abrogates ERK1/2 activation and nuclear translocation. In glioblastoma cells, ectopic LRRC4 expression competitively inhibited the interaction of endogenous mitogen-activated protein kinase (MEK) and ERK1/2. Mutation of the D domain decreased the LRRC4-mediated inhibition of MAPK signaling and its anti-proliferation and anti-invasion roles.

**Conclusions:**

Our results demonstrated that the D domain of LRRC4 anchors ERK1/2 in the cytoplasm and competitively inhibits MEK/ERK activation in glioma cells. These findings identify a new mechanism underlying glioblastoma progression and suggest a novel therapeutic strategy by restoring the activity of LRRC4 to decrease MAPK cascade activation.

**Electronic supplementary material:**

The online version of this article (doi:10.1186/s13045-016-0355-1) contains supplementary material, which is available to authorized users.

## Background

ERK (extracellular-signal-regulated kinase)/MAPKs (mitogen-activated protein kinases) are cytoplasmic serine/threonine kinases that transduce signals from the surface to the interior of the cell [1]. ERK1/2 is activated in response to multiple stimuli, including those that regulate cellular proliferation, differentiation, and survival [1]. Once activated, ERK1/2 disperses throughout the cell and phosphorylates a broad spectrum of substrates localized in different subcellular compartments, including the nucleus, and the cytoplasm [2]. The balance between the cytoplasmic and nuclear components of ERK1/2 signaling is critical for the biological outcomes resulting from ERK1/2 activation [3, 4]. Dysregulation of ERK/MAPK signaling is closely correlated with multiple diseases, including cancer, autoimmunity, and Alzheimer’s disease [5]. Increased ERK1/2 activity is found in majority of cancers and is a key event in tumor cell survival and proliferation [6]. The RAS/RAF/MEK/ERK/MAPK pathway has been reported to be activated in over 88 % of gliomas [7]. Both the RAS and RAF oncogenes are believed to promote initiation of human cancers by activating the ERK/MAPK signaling pathway [8, 9]. The aberrant nuclear accumulation of activated ERKs leads to tumor progression [10].

Leucine-rich repeat C4 protein (LRRC4), also known as netrin-G ligand-2 (NGL-2) [11], is a member of the leucine-rich repeat (LRR) superfamily [12]. It is predominantly localized to the postsynaptic side of excitatory synapses and is involved in early nervous system development and differentiation, especially synapse formation [11, 13–15]. LRRC4 regulates the formation of excitatory synapses through the recruitment of pre- and postsynaptic proteins [16], participates in the differentiation of neuron and glial cells, and promotes neurite outgrowth [17].

LRRC4 also is a tumor suppressor gene, and it is decreased in World Health Organization (WHO) grades II and III gliomas and absent in glioblastoma (WHO, grade IV) [18]. Promoter hypermethylation and miRNA dysregulation (miR-182, miR-381, and miR-185) have been identified as mechanisms underlying LRRC4 inactivation in glioma [19–21]. Enforced expression of LRRC4 reduced the activity of the Ras/c-Raf/ERK/MAPK and PI-3 K/AKT signaling pathways and inhibited cell proliferation and invasion in glioblastoma cells [22, 23].

Here, we demonstrated that amino acids 499-513 of the C-terminal of LRRC4 bind to ERK1/2 and constitute a reverse docking domain (D domain) with a consensus sequence: (R/K)_1-2_-(X)_2-6_-ØA-X-ØB (where ØA and ØB are Leu, Ile, or Val) [24, 25]. LRRC4 abolished ERK1/2 activation and inhibited ERK1/2 nuclear translocation through a direct interaction with ERK1/2 via the D domain, which inhibited ERK1/2 binding to MEK. Our results provided a novel regulatory mechanism for ERK1/2 activation and identified LRRC4 as a key modulator in ERK1/2 nuclear translocation.

## Results

### LRRC4 interacts with ERK1/2

**Fig. 1:**
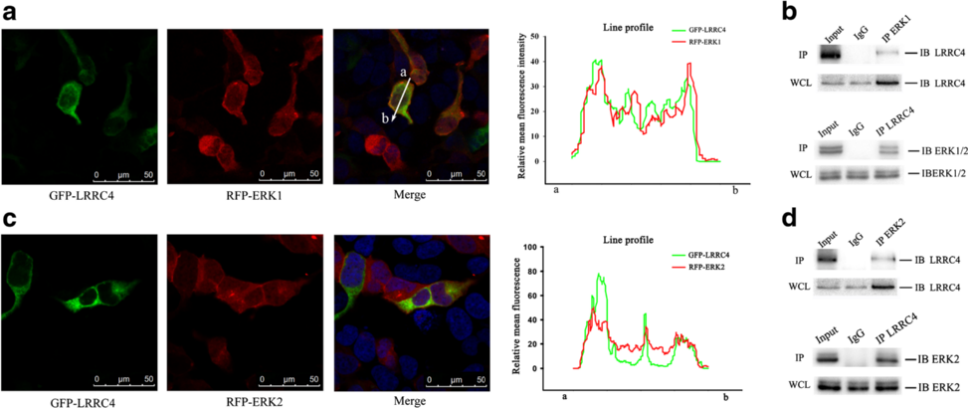
LRRC4 interacts with ERK1/2. **a** Confocal fluorescence microscopy of HEK293 cells co-transfected with GFP-LRRC4 (*green*) and RFP-ERK1 (*red*). The merged image shows co-localization of LRRC4 and ERK1 in the cytoplasm. The LRRC4 and ERK1 signals were measured by ImageJ software (scale bars, 50 μm). **b** HEK293 cells were transfected with pcDNA3.1(+)-LRRC4. Co-immunoprecipitation showed the interaction between LRRC4 and endogenous ERK1 in HEK293 cells. **c** Confocal fluorescence microscopy of HEK293 cells co-transfected with GFP-LRRC4 (*green*) and RFP-ERK2 (*red*). The merged image shows the co-localizations of LRRC4 and ERK2 in the cytoplasm. The LRRC4 and ERK2 signals were measured by ImageJ software (scale bars, 50 μm). **d** HEK293 cells were transfected with pcDNA3.1(+)-LRRC4. Co-immunoprecipitation showed the interaction between LRRC4 and endogenous ERK2 in HEK293 cells

Scansite 2.0 (version 2.0) software was used to screen for potential motifs or functional domains in LRRC4. When the high *stringency* criteria were used, a docking domain (D domain), an ERK-binding site, was found in the C-terminus of LRRC4. Therefore, we first determined whether LRRC4 co-localized with ERK1/2. HEK293 cells are good tools and useful for detecting the interaction of exogenous transfected proteins. We hypothesized that the interaction between LRRC4 and ERK1/2 is a natural existing state in normal human cells, and we used HEK293 cells to corroborate this hypothesis. We co-expressed green fluorescent protein (GFP)-LRRC4 with red fluorescent protein (RFP)-ERK1 in HEK293 cells and analyzed their co-localization by confocal fluorescence microscopy (Fig. 1a). In cells transfected with the GFP-LRRC4 and the RFP-ERK1/2 expression plasmids, ERK was co-localized with LRRC4 and was targeted almost exclusively to the plasma membrane with a perinuclear cytoplasmic distribution (Fig. 1a, merge). To determine whether LRRC4 and endogenous ERK1 could be co-immunoprecipitated from cells, a full-length LRRC4 protein expression vector was transfected in HEK293 cells. The endogenous ERK1 was co-immunoprecipitated with LRRC4 (Fig. 1b). Additionally, LRRC4 was co-immunoprecipitated with endogenous ERK1 (Fig. 1b). Moreover, LRRC4 and ERK2 also co-localized in the cytoplasm and plasma membrane of the cells (Fig. 1c, merge). LRRC4 and endogenous ERK2 co-immunoprecipitated with each other (Fig. 1d). Collectively, these results demonstrate that LRRC4 interacts with ERK1/2.

### A docking domain of the C-terminus of LRRC4 mediates the LRRC4-ERK1/2 binding and anchors ERK1/2 in the cytoplasm

**Fig. 2:**
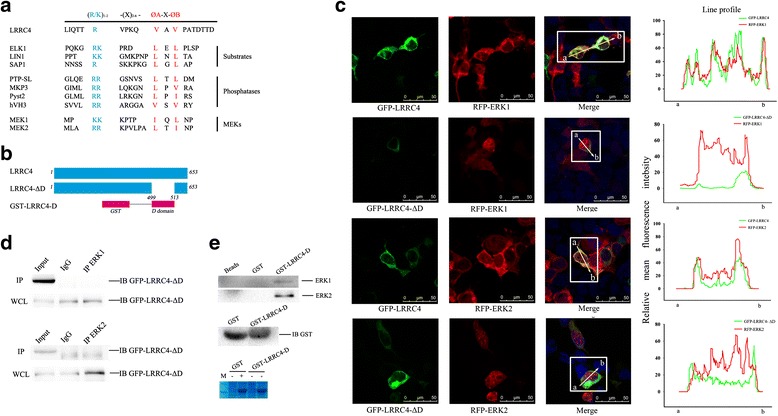
The D domain of LRRC4 mediates the LRRC4-ERK1/2 binding and anchors ERK1/2 in cytoplasm. **a** Alignments of the D domain sequences from LRRC4 and other proteins that contain the D domain, including MEKs, phosphatases, and substrates. **b** Schematic of the full-length LRRC4 protein, the LRRC4-ΔD mutant protein, and the GST-LRRC4-D fusion protein. **c** Confocal fluorescence microscopy of HEK293 cells co-transfected with different plasmids to assess the effect of D domain deletion on the co-localization of LRRC4 and ERK1/2. The merged image shows that ERK1 or ERK2 underwent nuclear translocation after the D domain of LRRC4 was deleted. **d** Co-immunoprecipitation showed that mutation of the D domain disrupted the interaction of LRRC4 and ERK1/2. (WCL: whole-cell lysate). **e** GST pull-down assays showed that the D domain of LRRC4 pulled down ERK1 and ERK2. Western blot and Coomassie blue staining analysis of whole-cell lysates (WCL) showed the expression of the GST fusion protein. (− : IPTG negative; + : IPTG positive)

The activity and specificity of MAP kinases must be tightly regulated to ensure proper integration of diverse biological stimuli and generation of appropriate cellular responses. The major mechanism to confer specificity and efficiency in MAP kinase signaling is through docking interactions between individual MAP kinases and their cognate activating kinases, inactivating phosphatases, scaffolding proteins, and substrates [24, 25]. A docking domain (D domain) sequence, (R/K)_1-2_-X_1-6_-Ø_A_-X-Ø_B_, was found in the C**-**terminus of LRRC4, which has been recognized in MAP kinase/ERK kinases, MAP kinase phosphatases (MKPs), scaffolding proteins, and MAP kinase substrates, where Ø_A_ and Ø_B_ are hydrophobic residues, such as Leu, Ile, or Val, and X is any amino acid (Fig. 2a).

To determine whether LRRC4 interacts with ERK1/2 through the D domain, we constructed an LRRC4 mutant (LRRC4-ΔD) with a deletion of the D domain in the full-length LRRC4 protein and a D domain fusion protein (GST-LRRC4-D) (Fig. 2b). We co-expressed GFP-LRRC4-ΔD with RFP-ERK1 or RFP-ERK2 in HEK293 cells and analyzed the transfected cells by confocal fluorescence microscopy. Compared with the co-distribution of wild type LRRC4 and ERK1/2, the cell distribution of GFP-LRRC4-ΔD was consistent with that of wild type LRRC4, but the subcellular distribution of RFP-ERK1 and RFP-ERK2 was altered when the D domain of LRRC4 was deleted. ERK1 and ERK2 were not only targeted to the plasma membrane and had a perinuclear cytoplasmic location but also translocated into the nucleus (Fig. 2c).

Almost no detectable nuclear ERK1/2 was observed in GFP-LRRC4 cells compared with that in GFP-LRRC4-ΔD cells. When the D domain of LRRC4 was deleted, the HEK293 cells retained a higher level of nuclear ERK1/2, despite detectable cytoplasmic ERK1/2 signals. The above observation showed that the D domain is critical for co-localization of LRRC4 and ERK1/2. After D domain deletion, ERK1 or ERK2 did not completely co-localize with LRRC4 in the cytoplasm, and the majority of ERK1 or ERK2 translocated to the nucleus. At the same time, after D domain deletion, the LRRC4-ΔD mutant did not co-immunoprecipitate with ERK1 or ERK2 (Fig. 2d). Similarly, a glutathione-S-transferase (GST) pull-down assay was performed with a fusion between ERK1/2 and a D domain (residues 499-513) of LRRC4. Wild-type ERK1 or ERK2 was precipitated with this GST-fused LRRC4-D peptide sequence (Fig. 2e). Overall, these data demonstrated that LRRC4 binds ERK1/2, and the D domain of the C**-**terminus of LRRC4 directly mediates the binding and anchoring of ERK1/2 in the cytoplasm.

### A conserved docking domain in ERK1/2 mediates the LRRC4-ERK1/2 binding and its cytoplasmic localization

**Fig. 3:**
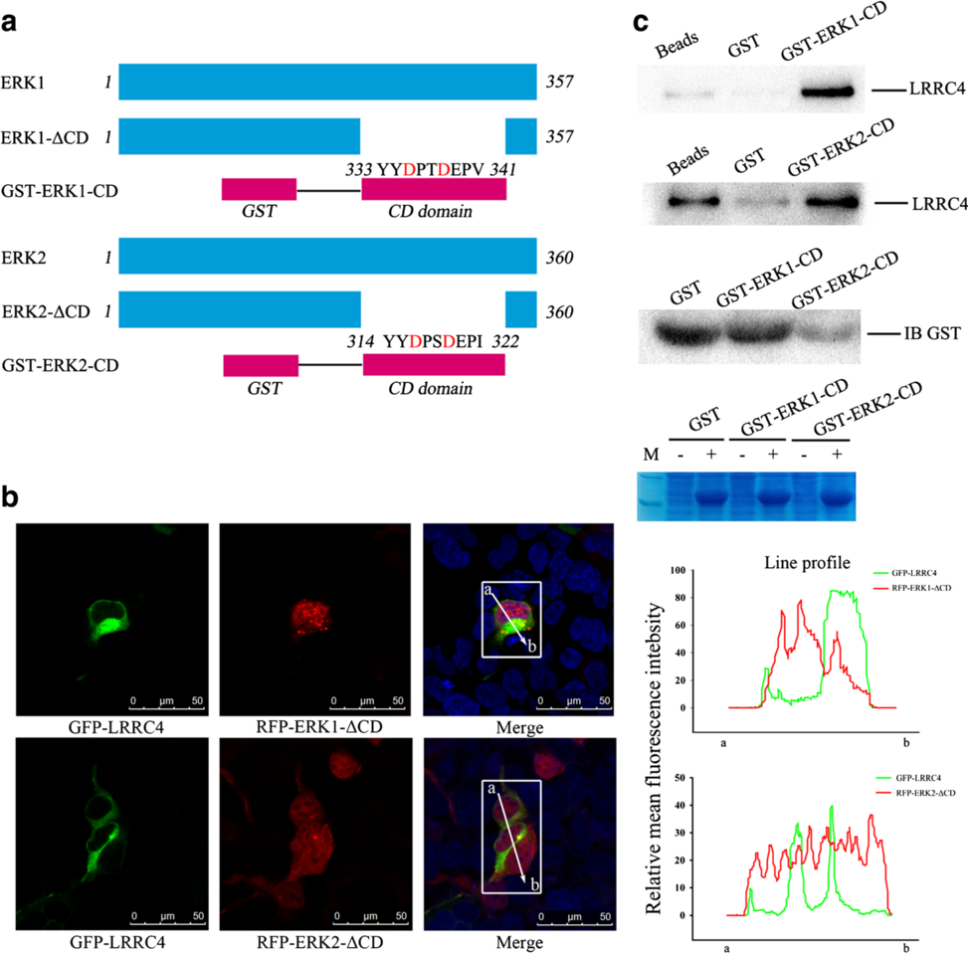
The CD domain of ERK1/2 mediates LRRC4-ERK1/2 binding and its cytoplasm localization. **a** Schematic of the ERK1 (ERK2) full-length protein, the ERK1 (ERK2)-ΔCD mutant protein, and the GST-ERK1 (ERK2)-D fusion protein. **b** Confocal fluorescence microscopy of HEK293 cells co-transfected with different plasmids to assess the effect of ERK1/2 CD domain deletion on the localization of LRRC4 and ERK1/2. The merged image shows that ERK1 or ERK2 underwent nuclear translocation after the CD domain of ERK1 or ERK2 was deleted. The signals were measured by ImageJ software. Scale bars, 50 μm. **c** GST pull-down assays showed that the CD domain of ERK1/2 pulled down LRRC4. Western blot and Coomassie blue staining analysis of whole-cell lysate (WCL) showed the expression of the GST fusion protein. (− : IPTG negative; + : IPTG positive)

A conserved docking domain (CD domain) that is present in the major members of the MAPK family, such as ERK, p38, and JNK, interacts with the D domain of the proteins. The CD domain is the near C-terminal region outside the catalytic domain of ERK1/2 [24]. We investigated whether ERK1/2 could interact with LRRC4 through its CD domain. We constructed a mutant with a deleted CD domain named ERK1-ΔCD or ERK2-ΔCD and a CD domain fusion protein (GST-ERK1-CD or GST-ERK2-CD) (Fig. 3a).

We co-expressed green fluorescent protein (GFP)-LRRC4 with red fluorescent protein (RFP) -ERK1-ΔCD or ERK2-ΔCD in HEK293 cells and analyzed the transfected cells by confocal fluorescence microscopy. After the CD domain of ERK1/2 was deleted, RFP-ERK1 or RFP-ERK2 did not co-localize with GFP-LRRC4. ERK1/2 was targeted to the plasma membrane and had a perinuclear cytoplasmic location and also translocated into the nucleus (Fig. 3b), but the CD domain deletion of ERK1/2 did not influence the subcellular localization of LRRC4. These observations indicated that the CD domain of ERK1/2 is critical for co-localization of LRRC4 and ERK1/2 in the cytoplasm. LRRC4 cannot anchor ERK1/2 in the cytoplasm without the CD domain of ERK1/2. At the same time, we also performed glutathione-S-transferase (GST) pull-down assays. Our results showed that both the CD domain of the GST-fused peptide of ERK1 and ERK2 can pull down the full-length LRRC4 protein (Fig. 3c). The data confirmed that LRRC4 binds ERK1/2 and anchors ERK1/2 in the cytoplasm via the D domain and CD domain, respectively.

### LRRC4 inhibits ERK1/2 activation and nuclear translocation via the D domain

**Fig. 4:**
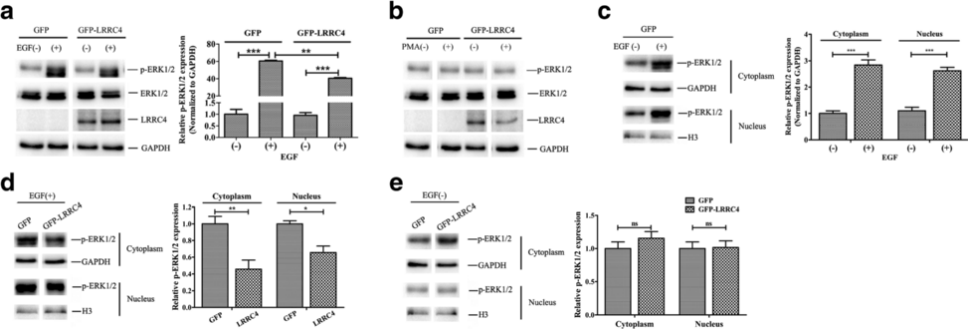
LRRC4 inhibits ERK1/2 activation. **a** HEK293 cells were transfected with GFP or GFP-LRRC4 followed by EGF (50 ng/mL) stimuli. Western blot analysis showed that LRRC4 inhibited the phosphorylation of ERK1/2 with EGF stimuli. **b** HEK293 cells were transfected with GFP or GFP-LRRC4 followed by PMA (1 μM) stimuli. Western blot analysis showed that PMA has no effect on the activation of ERK1/2 with or without LRRC4 in the HEK293 cells. **c** HEK293 cells expressing GFP were treated with EGF (50 ng/mL). Western blot analysis showed that EGF stimulation promoted increased expression of phosphorylated ERK1/2 in both the cytoplasm and nucleus with EGF stimuli. **d** HEK293 cells were transfected with GFP or GFP-LRRC4 followed by EGF (50 ng/mL) stimuli. Western blot analysis showed that LRRC4 inhibited the phosphorylation of ERK1/2 in both the cytoplasm and nucleus with EGF stimuli. **e** HEK293 cells were transfected with GFP or GFP-LRRC4 without EGF (50 ng/mL) stimulation. Western blot analysis showed that LRRC4 had no effect on pERK1/2 expression in both the cytoplasm and nucleus without EGF stimuli. The data represent the mean±SD of three replicates. *Bar* in the graph represents the s.e.m. Student’s *t* test, *NS* : no significant difference; *p < 0.05; **p < 0.01; ***p < 0.001

To investigate whether the interaction between LRRC4 and ERK1/2 affects ERK1/2 activation and nuclear translocation, we used EGF and PMA to stimulate the cells. HEK293 cells are LRRC4-negative and do not express LRRC4 with or without external stimuli (Fig. 4a, line 3, left). However, when HEK293 cells were transfected with GFP-LRRC4 (1 μg), EGF stimuli increased the expression of LRRC4 (Fig. 4a, line 3, right). Without LRRC4, EGF stimulation increased the expression of phosphorylated ERK1/2 (pERK1/2) (Fig. 4a, lines 1 and 2, left). Although LRRC4 had no effect on the expression of total ERK and pERK1/2 without EGF stimuli, LRRC4 inhibited EGF-induced expression of total ERK1/2 and pERK1/2 (Fig. 4a, lines 1 and 2, right). PMA had no effect on the activation of ERK1/2 with or without LRRC4 in the HEK293 cells (Fig. 4b).

Next, we isolated the cytoplasmic and nuclear fractions. Without LRRC4, EGF stimulation increased pERK1/2 in both the cytoplasm and nucleus, and there was no statistically significant difference (Fig. 4c). However, in the cells transfected with LRRC4, pERK1/2 was decreased in the cytoplasm and nucleus following EGF stimulation (Fig. 4d). Thus, LRRC4 inhibited EGF-induced pERK1/2 expression and nuclear translocation. Interestingly, without EGF, LRRC4 had no effect on the activation of ERK1/2 in the HEK293 cells (Fig. 4e). Taken together, LRRC4 expression reduced the ERK1/2 phosphorylation both in cytoplasm and nucleus after EGF treatment.

**Fig. 5:**
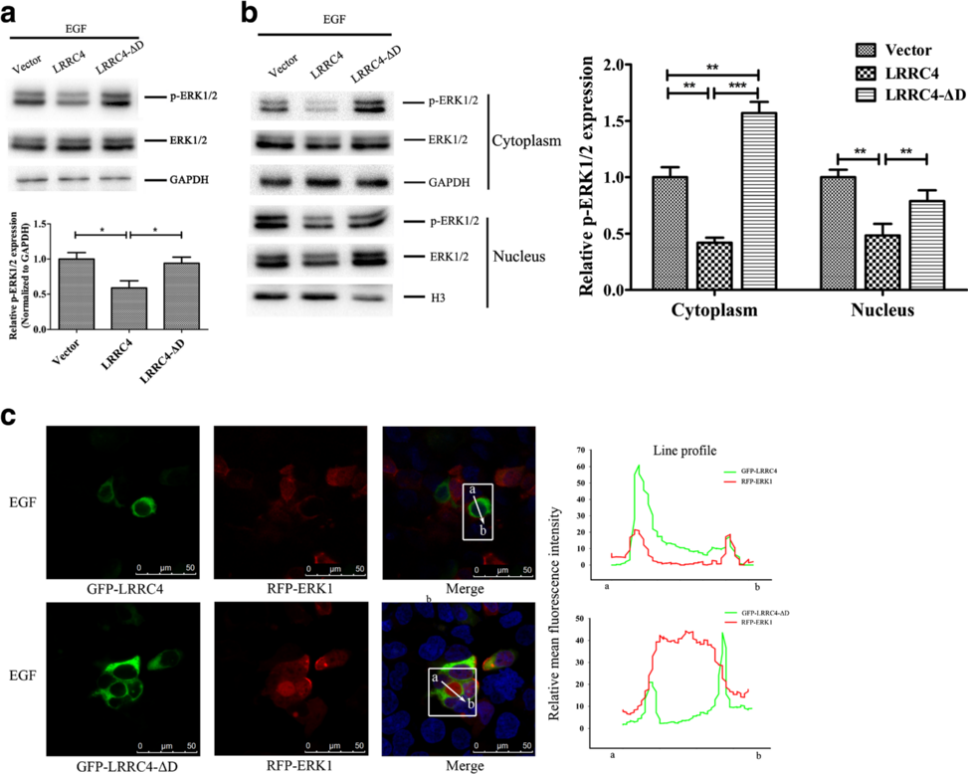
The D domain of LRRC4 abolished the activation and nuclear translocation of ERK1/2. **a** Western blot analysis showed that LRRC4 inhibited the phosphorylation of ERK1/2. After deletion of the D domain, the inhibitory effect of LRRC4 was weakened. **b** Western blot analysis showed that LRRC4 inhibited the phosphorylation of ERK1/2 both in the cytoplasm and nucleus. Deletion of the D domain in LRRC4 increased the phosphorylation level of ERK1/2 both in the cytoplasm and in the nucleus. **c** Confocal fluorescence microscopy of HEK293 cells co-transfected with different plasmids to assess the effect on localization of ERK1 and LRRC4 (LRRC4-ΔD) after EGF stimuli. The merged image shows that the translocation of active ERK1 to the nucleus was more significant after deletion of the D domain. The signals were measured by ImageJ software. (*Scale bars*, 50 μm.). The data represent the mean±SD of three replicates. *Bar* in the graph represents the s.e.m. One-way ANOVA, *p < 0.05; **p < 0.01; ***p < 0.001

Next, we investigated whether the D domain regulates ERK1/2 activation and nuclear translocation. In the presence of EGF, wild-type LRRC4 reduced the pERK1/2 expression, and when the D domain was deleted, this inhibition was weakened (Fig. 5a). As shown in Fig. 5b, in the presence of EGF, pERK1/2 in the nucleus (Fig. 5a, line 4, middle) was higher than that in the cytoplasm (Fig. 5a, line 2, middle). Compared to the vector group, wild-type LRRC4 inhibited pERK1/2 both in the cytoplasm and nucleus, and when the D domain of LRRC4 was deleted, the pERK1/2 level was increased in both the cytoplasm and nucleus. We hypothesized that LRRC4 inhibited EGF-induced pERK1/2 expression and nuclear translocation, and the D domain is the key motif for LRRC4 to inhibit the pERK1/2 expression and nuclear translocation. Confocal fluorescence microscopy also indicated that wild-type LRRC4 anchored ERK1/2 in the cytoplasm and inhibited the nuclear translocation of ERK1 in the presence of EGF. When the D domain was deleted, the mutant protein could not inhibit the nuclear translocation of ERK1, ERK1 did not completely co-localize with LRRC4 in the cytoplasm, and a majority of ERK1 translocated to the nucleus (Fig. 5c). These data further demonstrated that LRRC4 decreases ERK1/2 activation and prevents ERK translocation to the nucleus, and the D domain plays an important role in LRRC4 anchoring of ERK1/2 in the cytoplasm.

### LRRC4 prevents MEK binding to ERK1/2 in glioblastoma cells

**Fig. 6:**
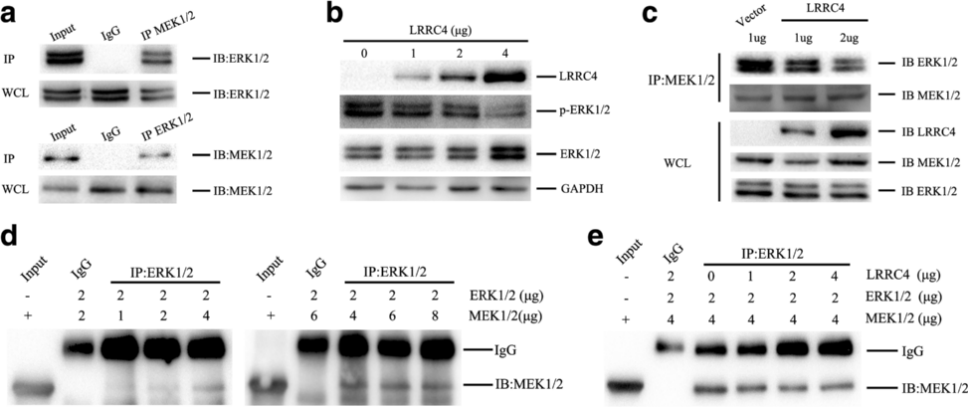
LRRC4 prevents MEK1/2 binding to ERK1/2 in U251 cells. **a** Co-immunoprecipitation of MEK1/2 and endogenous ERK1/2 in U251 cells. **b** U251 cells were transfected with different concentration of GFP-LRRC4. Western blot analysis showed that LRRC4 inhibits ERK1/2 activation, and the inhibition is dose-dependent. **c** U251 cells were transfected with GFP or GFP-LRRC4. The interaction of MEK 1/2 with ERK 1/2 in the presence of LRRC4 (different titrates) was analyzed by co-immunoprecipitation. **d** Co-immunoprecipitation of the purified MEK1/2 and ERK1/2 in vitro. The interaction of MEK1/2 and ERK1/2 became stronger with increasing MEK1/2 concentration. **e** The purified LRRC4 protein prevents MEK binding to ERK1/2 in vitro. The combination of the MEK1/2 and ERK1/2 was reduced with the increase in LRRC4 concentration

Since MEK1/2 binds to ERK1/2 and phosphorylates ERK1/2 through the D domain [26–28], we examined whether LRRC4 competes with MEK1/2 to bind ERK1/2 and prevents the ERK1/2 phosphorylation and nucleus translocation. We analyzed the ability of MEK1/2 to interact with ERK1/2 in U251 cells. Notably, MEK interacted with ERK1/2 in U251 cells (Fig. 6a). When U251 cells were transfected with a low dose of the LRRC4 plasmid (1 μg), LRRC4 affected the expression of total ERK1/2. When U251 cells were transfected with a high dose of the plasmid (4 μg), the expression of total ERK1/2 was also increased (Fig. 6a). With the increase in LRRC4 plasmids, the phosphorylation level of ERK1/2 diminished gradually (Fig. 6b).

Subsequently, we tested the effect of LRRC4 on the capacity of MEK1/2 binding to ERK1/2. As shown in Fig. 6c, following transfection with GFP-LRRC4, the ERK1/2 interaction with MEK1/2 was found to be diminished dramatically, suggesting that LRRC4 blocked the interaction of ERK1/2 with MEK1/2.

Then, we used the purified proteins to validate our conclusions in vitro*.* As shown in Fig. 6d, the interaction of MEK1/2 and ERK1/2 was stronger with increasing MEK1/2 concentration. Therefore, purified LRRC4 proteins were mixed in vitro*.* It was clear that the combination of MEK1/2 and ERK1/2 was reduced with increases in the LRRC4 concentration (Fig. 6e).

### LRRC4 abolishes ERK-mediated substrate activation and cell proliferation via the D domain

**Fig. 7:**
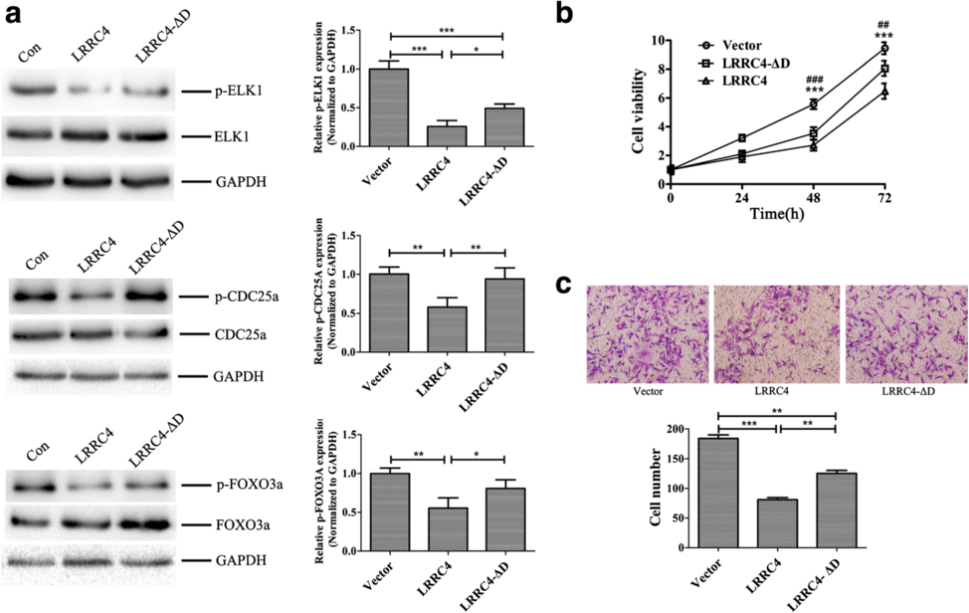
LRRC4 inhibits ERK-mediated activation of the downstream substrates to inhibit U251 cell proliferation via the D domain. **a** U251 cells were transfected with vector, LRRC4 or LRRC4-ΔD plasmid. Western blot showing the phosphorylation level of the ERK downstream substrates ELK1, FOXO3a, and CDC25a. **b** U251 cells were transfected with vector, LRRC4, or LRRC4-ΔD plasmids and were then subjected to CCK8 assays for the indicated times. ^*^LRRC4 vs vector, ^#^LRRC4-ΔD vs vector. ^***^ *p* < 0.001; ^##^ *p* < 0.01; ^###^ *p* < 0.001. **c** U251 cells were transfected with vector, LRRC4, or LRRC4-ΔD plasmid. Matrigel chamber invasion assay showing the invasion of U251 cells. The data represent the mean±SD of three replicates. *Bar* in the graph represents the s.e.m. One-way ANOVA, ^*^ *p* < 0.05; ^**^ *p* < 0.01; ^***^ *p* < 0.001; ^##^ *p* < 0.01; ^###^ *p* < 0.001

Upon activation and dimerization, ERK translocates to the nucleus, where it phosphorylates downstream substrates, such as the transcription factors ELK1 [29] and FOXO3a [30] and the tyrosine protein phosphatase CDC25a [31, 32]. Enforced LRRC4 expression inhibited the phosphorylation of ELK1, FOXO3a, and CDC25a, while deletion of D domain in LRRC4 restored the phosphorylation level of these proteins (Fig. 7a), suggesting that LRRC4 is a key inhibitor of ERK activation and decreased the phosphorylation level of ERK’s downstream substrates. Thus, the D domain is the key domain for LRRC4. We further assessed the effect of the D domain in LRRC4 on cell proliferation and invasion. Compared with wild-type LRRC4, deletion of the D domain weakened the LRRC4-mediated inhibition of cell proliferation and invasion (Fig. 7b, c). We also used U87 cells to assess the role of the D domain of LRRC4 in regulating the cell proliferation (Additional file 1: Figure S1b) and invasion (Additional file 1: Figure S1c) of GBM cells. Moreover, deletion of the D domain in LRRC4 restored the phosphorylation levels of ELK1, FOXO3a and CDC25a in U87 cells, and these results were consistent with those of the U251 cells.

## Discussion

The LRRC4 gene was first characterized from human chromosome 7q31-32 by our group [12, 18, 33]. Our studies indicated that LRRC4 is specifically expressed in brain tissue [12] and decreases in primary brain tumor biopsies, especially in gliomas (up to 87.5%) [12, 18]. The absence of LRRC4 expression contributes to late events in the pathogenesis of malignant glioblastoma. Studies have shown that the low expression of LRRC4 is due to the loss of heterozygosity on chromosome 7q32, promoter hypermethylation, and miRNA dysregulation in U251 cells [18, 34]. Ectopic LRRC4 expression inhibited glioblastoma cell proliferation and invasion in an ERK-dependent manner. Therefore, LRRC4 may act as upstream of ERK1/2 [18]. In this study, we found that LRRC4 binds with ERK1/2 and anchors ERK1/2 in the cytoplasm in HEK293 cells. The ectopic expression of LRRC4 abrogated the MEK1/2-ERK1/2 interaction in U251 cells. LRRC4 competitively inhibited the binding of ERK1/2 with MEK1/2 and prevented the phosphorylation of ERK1/2 and nuclear translocation, which further suppressed ERK-mediated activation of the downstream transcripts to inhibit cell proliferation and invasion.

These data further supported our previous hypothesis [18] that decreased LRRC4 accelerated the initiation and progression of glioblastoma [18, 34]. Following exogenous signaling stimulation, such as EGF, bFGF, IGF, and PDGF [35], the decreased LRRC4 also failed to block the ERK 1/2-MEK1/2 interaction, preventing MEK1/2 from sustaining activation for ERK1/2. Moreover, the epidermal growth factor receptor (EGFR) is overexpressed and/or mutated in at least 50% of GBM cases [36], which can further promote MEK/ERK/MAPK signal pathway activation. However, the MEK/ERK/MAPK pathway is one of the most frequently aberrantly activated signaling pathways in human cancers [37], including over 88% of gliomas [7].

Our studies also showed that LRRC4 binds to the CD domain of ERK1/2 via the D domain of the C-terminus. The D domain, also known as the kinase interaction motif (KIM), is a conserved amino acid sequence that has since been identified in nearly every MAPK regulatory protein, including MEKs, phosphatases, and substrates. The D domain is characterized by a consensus sequence: (R/K)1-2-(X)2-6-ØA-X-ØB (where ØA and ØB are Leu, Ile, or Val) [25]. The CD domain is the docking domain in the C-terminal lobe of MAPKs that determines binding specificity with substrate proteins [24, 38]. ERK1/2 and other MAPKs contain the CD domain, which includes aspartate residues 316 and 319 (labeled for ERK2) that are located on the opposite side of the TXY activation loop [24, 39] and mediate interactions with the D domains [39–41]. The D domain within Schnurri-3 mediated the interaction with ERK and inhibition of ERK activity and osteoblast differentiation [42]. Human scribble (hScrib) interacts with ERK through two D domain docking sites and decreases activation of ERK [43]. Ephrin-B3 (eB3), which contains a D domain, regulates synapse density by directly binding to ERK1/2 to inhibit postsynaptic Ras/MAPK signaling, and knockdown of eB3 resulted in a significant increase in the percentage of neurons with nuclear ERK1/2 localization [44]. In this study, we found that LRRC4 competitively binds the CD domain of ERK1/2 via the D domain, anchors ERK1/2 in the cytoplasm and prevents the activation induced by MEK.

Moreover, LRRC4 suppressed EGF-induced ERK1/2 phosphorylation and the activation of downstream transcription factors, such as ELK1, FOXO3a, and CDC25a, by preventing ERK1/2 translocation into the nucleus. ELK1 is a member of the Ets family of transcription factors and of the ternary complex factor subfamily [45, 46]. Activation of the ELK1 led to increased survival and proliferation following EGF stimulation in the U138 glioblastoma cells [47]. FOXO3a belongs to the forkhead family of transcription factors, which are characterized by a distinct forkhead domain [48, 49]. FOXO3a is an important regulator of proliferation and apoptosis in mantle cell lymphoma [49]. FOXO3a functions as a growth factor and promotes the proliferation of serum-deprived hepatocellular carcinoma cells [50]. CDC25a, a member of the CDC25 family of phosphatases, is required for progression from G1 to the S phase of the cell cycle [51, 52]. Interfering with CDC25a suppresses the growth and invasion in tumor cells [51, 53, 54]. Our results indicated that enforced LRRC4 expression prevents the activation of ERK downstream transcription factors to inhibit glioblastoma cell proliferation and invasion. The D domain is a critical domain for the LRRC4 anti-proliferation and anti-invasion activities.

## Conclusions

**Fig. 8:**
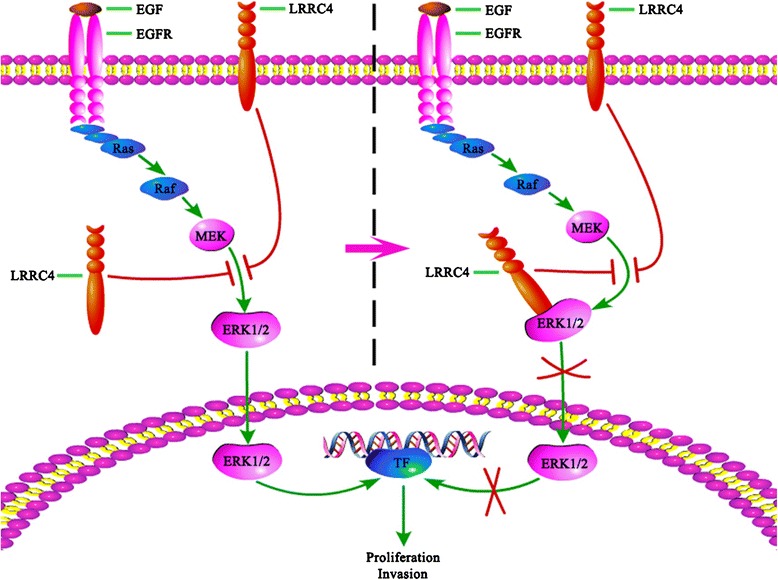
Schematic diagram of LRRC4 as a tumor suppressor in glioblastoma. The MEK/ERK/MAPK pathway is one of the most frequently aberrantly activated signaling pathways, and LRRC4 is an important tumor suppressor and has decreased expression in glioblastoma. Ectopic expression of LRRC4 abrogates the MEK1/2-ERK1/2 interaction. LRRC4 competitively inhibits the binding of ERK1/2 with MEK1/2 and prevents the phosphorylation of ERK1/2 and nucleus translocation. This further suppresses the ERK-mediated activation of the downstream transcripts to inhibit cell proliferation and invasion. In glioblastoma, the decrease or loss of LRRC4 failed to block the ERK 1/2-MEK 1/2 interaction and prevents MEK1/2 activation of ERK1/2

In conclusion, LRRC4 is an important tumor suppressor that directly interacts with ERK1/2 to disrupt the MEK1/2-ERK1/2 interaction and anchors ERK1/2 in the cytoplasm to mediate ERK1/2 inactivation, thus blocking ERK-mediated activation of the downstream substrates to suppress cell proliferation and invasion in glioblastoma cells (Fig. 8). Decreasing or silencing LRRC4 reduced its ability to inhibit the activation of ERK1/2 and nuclear translocation and then promoted tumorigenesis and progression of glioblastoma. These findings provided promising insights into developing novel cancer therapies by restoring the activity of LRRC4 to obstruct the MAPK cascade activation.

## Methods

### Cells culture and reagents

The human glioblastoma cell line U251 and human embryonic kidney (HEK) 293 cells were maintained in DMEM medium with high glucose and sodium pyruvate and supplemented with 10% fetal bovine serum and antibiotics (100 units/ml penicillin and 100 mg/ml streptomycin). Cells were incubated at 37 °C in a humidified atmosphere of 5% CO_2_. Antibodies against ELK-1 (BM0191) and phospho-ELK-1 (BM1095) were purchased from Abzoom Biolabs, Inc. (Dallas, TX, USA). Antibodies against MEK1/2 (#9122) and phospho-ERK1/2 (#9101) were purchased from Cell Signaling Technology (Beverly, MA, USA). Antibodies against ERK1 (sc-94), ERK2 (sc-154), and GAPDH (sc-32233) were from Santa Cruz Biotechnology (Santa Cruz, CA, USA). Antibodies against CDC25a (DP0870), phospho-CDC25a (DP0150), FOXO3a (DR1805), and phospho-FOXO3a (DP0315) were from UcallM Biotechnology Co., Ltd. (Wuxi, China). Antibodies against GFP (AG281), GST (AG768), and Histone H3 (AH433) were purchased from Beyotime Institute of Biotechnology (Jiangsu, China).

### Cell transfection

Cell transfection was performed using Lipofectamine 2000 (Invitrogen–Life Technologies, Carlsbad, CA, USA) using the manufacturer’s instructions.

### Confocal and image analysis

The cultured cells were plated on coverslips and transfected with plasmids. After transfection for 48 h, the cells were washed with PBS, fixed in 4% paraformaldehyde (PFA) at room temperature for 30 min, and incubated with 0.1% Triton X-100 in PBS for 10 min. Nuclear staining was performed with DAPI (Beyotime Institute of Biotechnology, Jiangsu, China). Coverslips were mounted and examined using a confocal laser scanning microscope (UltraView, Perkin Elmer, Cambridge, UK). Images were analyzed with ImageJ v1.440 (National Institutes of Health, Bethesda, MD).

### Immunoprecipitation

Lysates in RIPA buffer were incubated with antibody (0.3–0.6 mg) overnight at 4 °C with gentle rotation. A total of 80 ml of protein A Sepharose CL-4B beads (for rabbit immunoglobulin G [IgG]) or protein G Sepharose CL-4B beads (for mouse IgG) was added to the tubes and rotated at 4 °C for 2 h. Beads were precipitated by centrifugation at 16,000×*g* for 30 s and washed three times with cold RIPA buffer containing 150 mM NaCl. The pellets were resuspended in 2× Laemmli buffer and incubated at 100 °C for 5 min. The supernatants were used for western blot analysis.

### Western blotting

For total cell lysates, cells were lysed in lysis buffer that contained 25 mM Tris (pH 7.4), 2 mM NaVO_4_, 10 mM NaF, 10 mM Na_4_P_2_O_7_, 1 mM EGTA, 1 mM EDTA, and 1% NP-40. A protease inhibitor cocktail and PhosSTOP were added fresh to the lysis buffer before each experiment. Equivalent concentrations of protein (ranging 1–3 mg/ml) from each sample were placed in 1.5-ml tubes. Proteins were denatured in 1× Laemmli buffer by boiling at 100 °C for 5 min. The tubes were incubated at room temperature for 15 min before separation using SDS-PAGE. After resolving the proteins in SDS-PAGE, they were transferred onto a polyvinylidene fluoride (PVDF) membrane. The membrane was blocked in Tris-buffered saline (TBS; pH 7.4) with the 10% blocking reagent provided with the BM Chemiluminescence Blotting Substrate (POD) assay system for 1 hr, followed by incubation with primary antibody in TBS-Tween (TBST; pH 7.4) with 5% blocking reagent at 4 °C overnight. After the incubation, the membrane was washed three times in TBST, followed by incubation with secondary antibody in TBST 10% blocking reagent for 1 h, and washed again in TBST (three times for 20 min). Immunoblots were developed using ChemicalDoc^TM^ XRS+ (Bio-Rad, Berkeley, CA, USA). The intensity of the protein bands was quantified using Quantity One software (Bio-Rad, Berkeley, CA, USA).

### GST pull-down assay

Bacterial cells were lysed using the following buffer: 20 mmol/L Tris-Cl, 150 mmol/L NaCl, 2 mmol/L EDTA, 0.5% NP40, pH 7.5. To determine the interaction between the D domain (CD domain) and ERK1/2 (LRRC4), bacterial lysates containing GST-D domain were incubated with glutathione-Sepharose 4B beads at 4 °C for 1 h. The beads were washed and incubated with bacterial cell lysates containing ERK1/2 (LRRC4), allowing the interaction between GST-D domain (GST-CD domain) and ERK1/2 (LRRC4). After washing, the GST-D domain and the bound ERK1/2 were eluted from the beads and subjected to electrophoresis.

### Nuclear protein extraction

For nuclear protein extraction from cells in 6 cm plates, cells were removed from the dishes by scraping with 300 ml of cytoplasmic lysis buffer (10 mM HEPES [pH 7.5], 2 mM MgCl_2_, 1 mM EDTA, 1 mM EGTA, 10 mM KCl, 10 mM NaF, 0.1 mM Na_3_VO_4_, protease inhibitor cocktail, and PhosSTOP). Following 15 min of incubation on ice, 25 ml of 10% NP-40 was added and vortexed for 10 s. The cells were centrifuged for 1 min at 16,000×*g*, and supernatants were collected to obtain the cytoplasmic fractions. The pellets were resuspended in 200 ml of nuclear lysis buffer (25 mM HEPES [pH 7.5], 500 mM NaCl, 10 mM NaF, 10% glycerol, 0 .2% NP-40, 5 mM MgCl_2_, and 10 mM dithiothreitol [DTT]). RIPA buffer was used instead of nuclear lysis buffer for immunoprecipitation experiments. The suspension was incubated on ice for 30 min. During this incubation, lysates were vortexed every 10 min. Finally, cells were centrifuged for 10 min at 16,000×*g* to obtain nuclear proteins. For nuclear extraction from liver tissues, 50 mg of liver tissue was cut in small pieces and washed once with ice-cold PBS. Nuclear proteins were isolated using a commercially available kit from Pierce according to the manufacturer’s instructions, with no modifications.

### CCK8 assay

Cell viability was determined with CCK8 assays. Briefly, 2000 cells/well were seeded into 96-well plates and were treated by plasmid vector transient transfection, and the absorptions of the cells were measured using a CCK8 kit (Beyotime Institute of Biotechnology, Jiangsu, China) according to the manufacturer’s instruction at different indicated time points. Data were derived from three separate experiments with four replicates each time.

### Matrigel chamber invasion assay

Diluted matrigel (BD Biosciences) was added to the upper well of the Transwell chamber (Corning Inc., Corning, NY) and reconstituted for 1 h at 37 °C. The cells were starved overnight in serum-free medium and resuspended at a concentration of 2.5×10^5^ cells/ml in serum-free medium containing 0.1% bovine serum albumin. Then, 0.2 ml cell suspension was added to the top of each well, and a 10 mg/ml fibronectin solution was added to the bottom well of the chamber as a chemoattractant. After 36 h, the cells that had not invaded were removed from the upper surface of the filters using a cotton swab. The cells that had invaded to the lower surface of the filter were fixed with methanol and stained with H&E, and 5 random fields (409) were counted. The data are expressed as the mean value of cells per field in triplicate in two independent experiments.

### Statistical analysis

All experiments were performed three times, and the data were analyzed with GraphPad Prism 5 (La Jolla, CA, USA). Differences between the variables of the groups were tested using Student’s *t* test or one-way ANOVA, using the SPSS 15.0 program. A *p*-value of <0.05 was statistically significant.

## Additional file

Additional file 1: Figure S1. LRRC4 inhibits ERK-mediated activation of the downstream substrates to inhibit U87 cell proliferation via the D domain. (TIF 14723 kb)

## Abbreviations

CD domain: Conserved docking domain
CDC25a: Cell division cycle 25 homolog A
D domain: Docking domain
EGF: Epidermal growth factor
ERK1/2: Extracellular regulating kinase 1/2
FGF: Fibroblast growth factor
FOXO3a: Forkhead box O3A
GFP: Green fluorescent protein
GST: Glutathione S-transferase
HEK293: Human embryonic kidney 293 cells
IGF: Insulin-like growth factor 1
LRRC4: Leucine rich repeat containing 4
MAPK: Mitogen-activated protein kinase
MEK: Mitogen-activated protein kinase kinase
NGL-2: Netrin-G ligand-2
PDGF: Platelet-derived growth factor
PMA: Phorbol 12-myristate 13-acetate
RFP: Red fluorescent protein
