# Supplementary figures and images for: The D Domain of LRRC4 anchors ERK1/2 in the cytoplasm and competitively inhibits MEK/ERK activation in glioma cells
Source: J Hematol Oncol. 2016 Nov 25;9:130. doi: 10.1186/s13045-016-0355-1 (PMC5123285; doi:10.1186/s13045-016-0355-1)

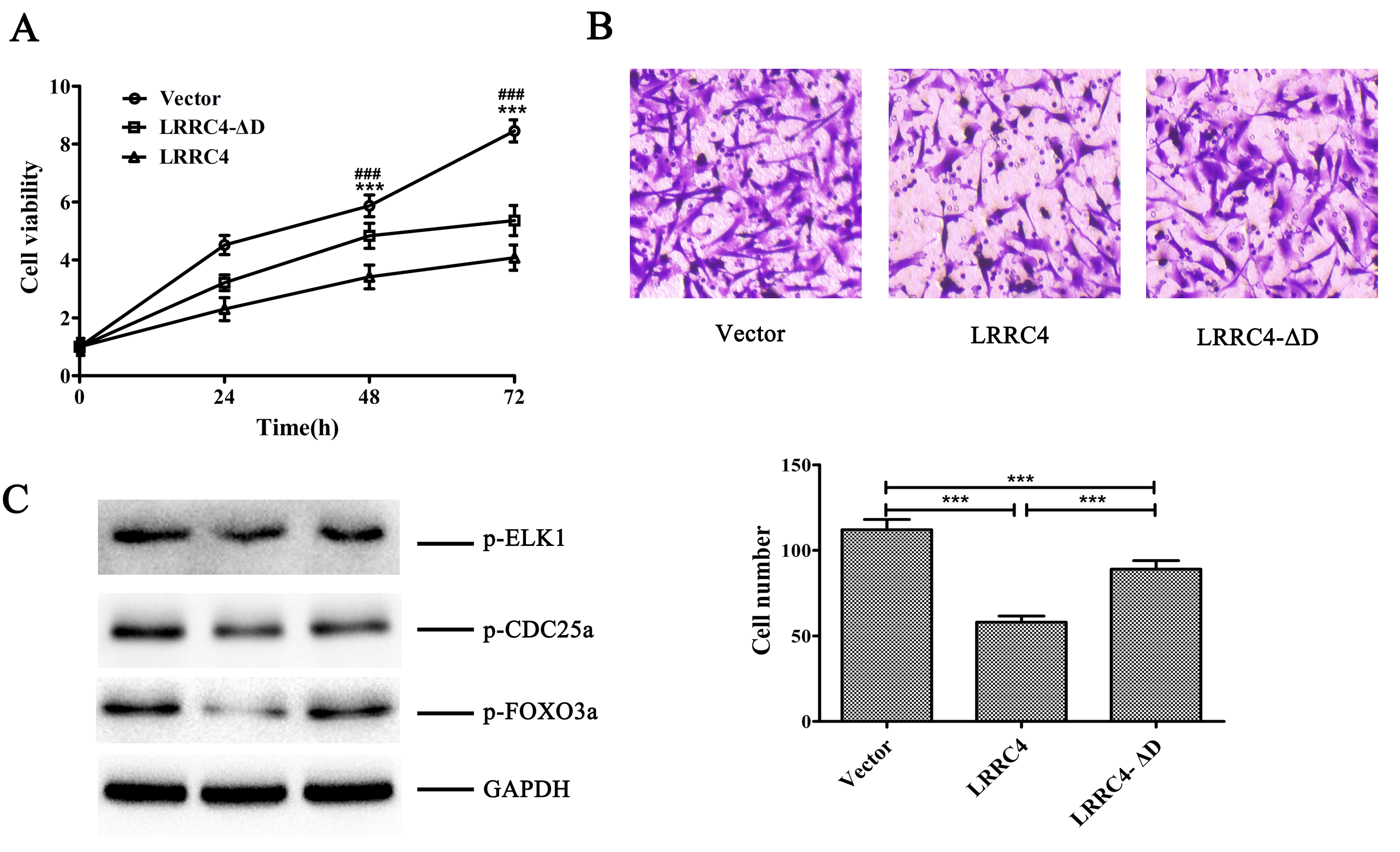

Supplement: Additional file 1: Figure S1. — LRRC4 inhibits ERK-mediated activation of the downstream substrates to inhibit U87 cell proliferation via the D domain. (TIF 14723 kb) [file 13045_2016_355_MOESM1_ESM.tif]
